# Update of the Brazilian consensus recommendations on Duchenne muscular dystrophy

**DOI:** 10.1055/s-0043-1761466

**Published:** 2023-03-14

**Authors:** Alexandra Prufer de Queiroz Campos Araujo, Jonas Alex Morales Saute, Clarisse Pereira Dias Drumond Fortes, Marcondes Cavalcante França Jr, Jaqueline Almeida Pereira, Marco Antonio Veloso de Albuquerque, Alzira Alves de Siqueira Carvalho, Eduardo Boiteux Uchôa Cavalcanti, Anna Paula Paranhos Miranda Covaleski, Simone Chaves Fagondes, Juliana Gurgel-Giannetti, Marcus Vinicius Magno Gonçalves, Alberto Rolim Muro Martinez, Antônio Rodrigues Coimbra Neto, Flavio Reis Neves, Anamarli Nucci, Ana Paula Cassetta dos Santos Nucera, Andre Luis Santos Pessoa, Marcos Ferreira Rebel, Flavia Nardes dos Santos, Rosana Herminia Scola, Cláudia Ferreira da Rosa Sobreira

**Affiliations:** 1 Universidade Federal do Rio de Janeiro, Faculdade de Medicina, Rio de Janeiro RJ, Brazil.; 2 Universidade Federal do Rio Grande do Sul, Faculdade de Medicina, Porto Alegre RS, Brazil.; 3 Instituto de Puericultura e Pediatria Martagão Gesteira, Equipe de Pesquisa em Doenças Neuromusculares, Rio de Janeiro RJ, Brazil.; 4 Universidade Estadual de Campinas, Faculdade de Ciências Médicas, Campinas SP, Brazil.; 5 Universidade Federal do Rio de Janeiro, Faculdade de Fisioterapia, Rio de Janeiro RJ, Brazil.; 6 Universidade de São Paulo, Hospital das Clínicas, São Paulo SP, Brazil.; 7 Centro Universitário da Faculdade de Medicina do ABC, Santo André SP, Brazil.; 8 Hospital Sarah Kubitschek, Unidade Lago Norte, Brasília DF, Brazil.; 9 Associação de Assistência à Criança Deficiente, Ambulatório de Doenças Neuromusculares, Recife PE, Brazil.; 10 Hospital das Clínicas de Porto Alegre, Laboratório do Sono, Porto Alegre RS, Brazil.; 11 Faculdade de Medicina, Universidade Federal de Minas Gerais, Belo Horizonte MG, Brazil.; 12 Faculdade de Medicina, Universidade da Região de Joinville, Joinville SC, Brazil.; 13 Serviços de Doenças Neuromusculares, Universidade Estadual de Campinas, Campinas SP, Brazil.; 14 Universidade Estadual do Ceará, Faculdade de Medicina, Fortaleza CE, Brazil.; 15 Universidade Federal do Paraná, Faculdade de Medicina, Curitiba PR, Brazil.; 16 Universidade de São Paulo, Faculdade de Medicina de Ribeirão Preto, Ribeirão Preto SP, Brazil.

**Keywords:** Muscular Dystrophy, Duchenne, Consensus, Practice Guideline, Diagnosis, Genetic Testing, Steroids, Rehabilitation, Drug Therapy, Standard of Care, Drug Development, Distrofia Muscular de Duchenne, Consenso, Guia de Prática Clínica, Diagnóstico, Testes Genéticos, Esteroides, Reabilitação, Tratamento Farmacológico, Padrão de Cuidado, Desenvolvimento de Medicamentos

## Abstract

In the last few decades, there have been considerable improvements in the diagnosis and care of Duchenne muscular dystrophy (DMD), the most common childhood muscular dystrophy. International guidelines have been published and recently reviewed. A group of Brazilian experts has developed a standard of care based on a literature review with evidence-based graded recommendations in a two-part publication. Implementing best practice management has helped change the natural history of this chronic progressive disorder, in which the life expectancy for children of the male sex in the past used to be very limited. Since the previous publication, diagnosis, steroid treatment, rehabilitation, and systemic care have gained more significant insights with new original work in certain fields. Furthermore, the development of new drugs is ongoing, and some interventions have been approved for use in certain countries. Therefore, we have identified the need to review the previous care recommendations for Brazilian patients with DMD. Our objective was to create an evidence-based document that is an update on our previous consensus on those topics.

## Resumo

Nas últimas décadas, houve progressos significativos no diagnóstico e no tratamento da distrofia muscular de Duchenne (DMD), considerada a distrofia muscular mais comum na infância. Diretrizes internacionais foram publicadas e revisadas recentemente. Um grupo de especialistas brasileiros desenvolveu um padrão de atendimento baseado em revisão de literatura, com recomendações graduadas pautadas em evidências compiladas em uma publicação dividida em duas partes. A implementação de melhores práticas de manejo ajudou a modificar a história natural desta doença crônica, progressiva, que, no passado, oferecia uma expectativa de vida muito limitada para crianças do sexo masculino. Desde a publicação desse consenso anterior, o diagnóstico, o tratamento com esteroides, a reabilitação e os cuidados sistêmicos ganharam novas possibilidades a partir da divulgação dos resultados de trabalhos originais em algumas dessas áreas. Além disso, as pesquisas e o desenvolvimento de novos fármacos estão em andamento, e algumas intervenções já foram aprovadas para uso em determinados países. Nesse contexto, identificamos a necessidade de rever as recomendações anteriores sobre o manejo dos pacientes brasileiros com DMD. Nosso objetivo principal foi elaborar uma atualização baseada em evidências sobre esses tópicos do consenso.

## INTRODUCTION

In the last few decades, there have been considerable improvements in the diagnosis and care of Duchenne muscular dystrophy (DMD), the most common childhood muscular dystrophy. International guidelines have published and recently reviewed recently. 1 2 A group of Brazilian experts has developed a standard of care based on a literature review with evidence-based graded recommendations in a two-part publication. 3 4 Implementing best practice management has helped to change the natural history of this chronic progressive disorder, in which the life expectancy for children of the male sex in the past used to be very limited. 5 6

Since the previous publication, diagnosis, steroid treatment, rehabilitation, and systemic care have gained more significant insights with new original work in certain fields. Furthermore, the development of new drugs is ongoing, and some interventions have been approved for use in certain countries. Therefore, we have identified the need to review the previous care recommendations for Brazilian patients with DMD. Our objective was to create an evidence-based document that is an update on our previous consensus on those topics.

## METHODS

The present working group was composed of members of the Neuromuscular Disorders Department of the Brazilian Academy of Neurology who were invited and accepted the work timeline, and medical doctors and physical therapists currently involved in DMD care and/or research.

We performed a search for articles published in last five years on PubMed with Mesh search using the keyword *muscular dystrophy, Duchenne* ” with any one of the following keywords alone or in combination: *practice guideline* ; *diagnosis* ; *genetic testing* ; *newborn screening* ; *glucocorticoids* ; *therapeutics* ; *therapy* ; *physical therapy modalities* ; *exercise* ; *rehabilitation* ; *noninvasive ventilation* ; *cognition* ; *quality of life* ; *orthotic devices* ; *muscle stretching exercises* ; *tracheostomy* ; *vital capacity* ; *respiratory function tests* ; *cardiomyopathies* ; *heart failure* ; *nutrition disorders* ; *nutritional support* ; *bone health* ; *drug therapy* ; *ataluren* ; *eteplirsen* ; *golodirsen* ; *casimersen* ; *viltolarsen* ; *exon skipping* ; *readthrough* ; and *gene therapy* . Perspective topic has also used those web sites: ClinicalTrials.gov, ANVISA, FDA, and EMA websites were included in the search.

Each working group searched for newly-published literature starting with questions ( Table 1 ) from the Delphi technique approach, the same method used in our previous work. 3 4 A total of 136 articles published since 2016 were listed and reviewed. Publications that were more relevant and those with higher levels of evidence were included, and the redundant ones were excluded. Therefore, each working group selected information for the present revision not explored in our last publication. 3 4

**Table 1:** Initial steps for the update

Topic	Question
**Diagnosis**	Any new genetic tests? Any modification to the previous practice guideline? Methods to increase early diagnosis?
**Steroid therapy**	New publications comparing regimens and/or drugs? Any modification on starting age? On conducting through different stages of DMD? Alternative drug?
**Rehabilitation**	New publications on strength exercises, orthoses, or pulmonary care recommendations? Definition of assessments in clinical practice? Definition of DMD stages to implement each recommendation?
**Systemic care**	New publications on heart assessment and protection? On bone health and orthopedic intervention? New recommendation on vaccines?
**New perspectives**	Critical review on approved specific drugs after phase 3 clinical trials. Any other new perspective?

Abbrevation: DMD, Duchenne muscular dystrophy.

Discussion sessions of the working groups followed, with new rating for the level of evidence and class of recommendation, as previously described. 3 4 The statements developed by each working group were submitted to all members on an anonymous voting system of five different Likert scale options (1. strongly agree; 2. agree; 3. neither agree nor disagree; 4. disagree; and 5. strongly disagree).

## DIAGNOSIS

Performing an accurate diagnosis is the starting point for the standard of care for DMD. Diagnostic confirmation enables the performance of proper interventions and provides educational and support information and adequate genetic counseling to empower families. The investigation will often start with clinical suspicion by pediatricians, general practitioners, and other health care professionals, who must be aware of the condition and its diagnosis. Ideally, an expert in neuromuscular diseases should order and interpret confirmatory studies; however, due to the greater availability of genetic testing for DMD in Brazil in recent years, in the current days, it is expected that other physicians take this approach.

The present consensus recommends that any physician who orders a genetic analysis for DMD should be aware of the appropriate tests, should know how to interpret the results, and should provide adequate posttest counseling or seek input for this step from a medical geneticist or expert in neuromuscular diseases (level of evidence: 5; class of Recommendation: D; expert opinion: 69.2% strongly agree; 23.1% agree). 7

### Diagnostic suspicion

The criteria for DMD diagnosis suspicion have remained the same since the previous Brazilian consensus. 3 The diagnosis of DMD should be considered in any boy, irrespective of family history, with any of the following aspects: 1) proximal weakness with onset between the ages of 2 and 5 years; 2) psychomotor developmental delay, including delay in the achievement of motor milestones or speech acquisition, intellectual disability, or autism spectrum; 3) calf hypertrophy; 4) marked increase in creatine kinase (CK); or 5) incidental finding of elevated levels of transaminases. If any of these criteria are present, a screening evaluation of the CK levels should be ordered and confirmed in a second sample assay. 3

### Diagnostic confirmation

The diagnostic confirmation strategies were narrowed from the previous Brazilian consensus to make them closer to the current clinical practice. We reinforce that testing for pathogenic variants in the *DMD* gene, will always be necessary, even if the diagnosis has been confirmed by the absence of dystrophin protein expression on muscle biopsy, to provide accurate information for genetic counseling and enable the detection of mutation carriers. 3 Considering the most frequent types of pathogenic variants in *DMD* , multiplex ligation-dependent probe amplification (MLPA), which detects large deletions and duplications, is the first-line confirmatory genetic testing. Importantly, physicians should be aware that detecting a single exon deletion on MLPA may be a false positive result due to point mutation or polymorphisms in the probe binding site. Thus, an orthogonal second test, such as Sanger sequencing of the involved exon or next-generation sequencing (NGS) of *DMD* , must be performed to complement the test and confirm the diagnosis in this scenario. 3 If the deletion/duplication testing is negative, then *DMD* sequencing should look for small-scale pathogenic variants, and the NGS is the second-tier method of choice. Suppose both large deletions/duplications and sequencing analysis of DMD are negative. In that case, a muscle biopsy with the immunohistochemistry and immunoblotting for dystrophin interpreted by an experienced neuromuscular pathologist should be performed. Additionally, muscle biopsy with immunohistochemistry may be required to support the diagnosis when variants of unknown significance are found in *DMD* sequencing. 3

For more details about evidence-based genetic testing for *DMD* and the use of imaging and neurophysiological studies for DMD diagnosis, we refer to the previous consensus. 3 The detection of adult female carriers of DMD should be performed with molecular testing; for details about evidence-based genetic testing for carriers, we refer to the previous consensus. 3

### Update on NGS of DMD

For the diagnosis of DMD, NGS of *DMD* can also be used as the first genetic study. In a recent study, 8 the NGS detected all large deletions of the gene and 50% of large duplications, resulting in a high detection rate (level of evidence: 4; class of recommendation: C). In the clinical practice, NGS of *DMD* can be performed as the first-tier confirmatory strategy for diagnosis only in laboratories that have validated algorithms to detect copy number variations of this gene from NGS data. Due to false-negative results for large duplications with NGS, 8 MLPA should be performed if negative results are found. Additionally, the greater availability of NGS technologies also made it possible to use panels of genes, including *DMD* and other genes related to inherited neuromuscular disorders, or even exome sequencing, 9 instead of evaluating only *DMD* , as first- or second-tier diagnostic approaches. If such studies are available, they will have the advantage of disclosing with a single test the diagnosis of DMD and other genetic neuromuscular disorders without the need for further genetic or histopathological studies. This consensus considers the use of NGS of *DMD* (in panels with single or multiple genes) as the first-tier genetic testing for DMD as an alternative to the sequential strategies of MLPA plus NGS of *DMD* (level of evidence: 4; class of recommendation: C; expert opinion: 30.8% strongly agree; 53.8% agree). 8 9 The costs of tests, the local availability of the exam, and the discussion with the families should guide the clinician in deciding the best diagnostic strategy.

For more information about evidence-based prenatal and preimplantation diagnosis for DMD, we refer to the previous consensus. 3

### Neonatal screening for Duchenne muscular dystrophy

The studies on neonatal screening for DMD started in the mid-1970s, with CK measurement and genetic testing being the main techniques. 9 10 11 12 The measurement of CK on filter cards has demonstrated adequate sensitivity to diagnose DMD in the neonatal period, although false-negative results have been reported in populational studies with longer follow-up. 11 The cut-offs for a positive screening, which vary greatly among programs, and the sample time have led to important variations in test specificity. 10 11 12 Of note, although infrequent, screening based on CK elevation has also identified patients with other genetic muscle diseases such as limb-girdle muscular dystrophies, some of them with late onset. 12 In general, the screening programs performed a second blood CK measurement by the age of 6 to 8 weeks to confirm the findings on filter cards and to perform confirmatory genetic studies. 10 11 However, collection strategies in the first 24 to 48 hours of life, with molecular confirmation in the same sample, have been validated. 12

A recent study 9 in China has shown that NGS could also be used as a neonatal screening method. Some studies 13 14 have sought to assess the impact of newborn screening for DMD on the perception of parents who had children with DMD identified by these programs. In general, the parents' perception was that early diagnosis was positive, mainly because it enabled them to prepare emotionally to deal with the condition. 13 14 Notably, these parents considered that screening should be optional rather than mandatory. 13 15 16 Parental perceptions seem to be influenced by cultural factors. They may vary in different countries, with some parents questioning the infrastructure available to meet the demands of a patient with an early diagnosis of DMD. 17 Most experiences have shown that the possible risks and damages of screening programs are minimal, even for patients who have had children with transient elevation of CK. The mother-infant relationship did not seem to have been affected, and some families have modified their reproductive plans after the screening. 14

On the other hand, the perception of health professionals differs from that of parents of patients with DMD. In a study carried out in the United States, 18 only 1/4 of pediatricians, whether experts in DMD care or not, favored neonatal screening for DMD. Conversely, most professionals favored testing groups at higher risk for the disease. 18

Technically, CK dosage (level of evidence: 2B; class of recommendation: B) 10 11 12 and genetic analysis of *DMD* can be used for DMD neonatal screening (level of evidence: 2B; class of recommendation: B). 9 The standards for the inclusion of diseases in the neonatal screening program in general and at the public level in Brazil include the frequency of the disease, short period between birth and onset of symptoms, and availability of disease-modifying therapies. Considering that the disease-modifying therapies indicated before the onset of symptoms are not available for DMD, and considering the complexity of implementing screening by CK dosage in a country of continental size, with a significant number of false-positive results and divergence of cutoff points, and the possibility of disclosing a diagnosis of late-onset diseases in the neonatal period, it is currently not possible to recommend widespread DMD screening in the National Neonatal Screening Program in Brazil (expert opinion: 38.5% agree; 23.1% neither agree nor disagree). However, we emphasize that, given the possible emergence and availability of new therapies for the treatment of DMD, this scenario may change in the coming years.

### Screening the higher-risk population

The early investigation of children with developmental (motor, cognitive, or language) delay and the incidental identification of increased CK and/or transaminase levels and in cases with a positive family history of DMD is highly recommended to establish an early diagnosis. 3 Early diagnosis will avoid invasive or inadequate tests in these situations, enabling an early treatment plan with a multidisciplinary team. Besides, it would define the best time to start corticosteroid therapy and enable earlier genetic counseling for the family, promoting their empowerment and helping in their reproductive and life planning. Thus, the present consensus recommends early screening for DMD in a higher-risk population with dosage of CK levels (level of evidence: 5; class of recommendation: D; expert opinion: 61.5% strongly agree; 30.8% agree).

## STEROID THERAPY

Corticosteroids (CSs) have been the mainstay of pharmacological treatment in DMD patients. 19 The first clinical trial was published in 1989, 20 and it showed clear benefits for DMD patients by modifying the natural history of the disease. Since then, the short- and long-term positive effects of CSs in DMD became evident: increased muscle strength, increased pulmonary and cardiac function, and, later, increased overall survival. 21 The following are the reasons for the main recommendation for CSs: steroid use is mandatory from the age of 5 years on (expert opinion: 54.5% strongly agree; 36.4% agree). Corticosteroids should be prescribed for all DMD patients (as early as 2 to 5 years of age) and even after loss of ambulation (expert opinion: 54.5% agree; 27.3% disagree). Patients on steroids should be reassessed every 3 to 4 months (expert opinion: 36.4% strongly agree; 45.5% agree). Steroids are indicated for wheelchair users (expert opinion: 54.5% strongly agree; 36.4% agree). Discontinuation of the steroid is only indicated in the presence of adverse events that cannot be controlled with drugs and/or lifestyle changes (expert opinion: 72.7% strongly agree; 27.3% agree).

Despite the notable positive change in the natural history of DMD, several questions remain unanswered: which is the best age to start CSs? When should CSs be discontinued? What is the best regimen to prevent or manage the side effects of CSs? Since the publication of the previous edition of the Brazilian DMD Guideline, 3 head-to-head comparisons of different types, doses, regimens, and studies looking at the precise moment to start or discontinue CS therapy are still sparse without conclusive or definitive evidence. As a practical approach, Table 2 from the previous guidelines still stands as an overview of the leading CS options:

**Table 2:** Different corticosteroid regimens for Duchenne muscular dystrophy patients, their pros and cons, and the suggested follow-up schedule

Drug (dose- regimen)	Favorable features	Disadvantages	Follow-up schedule*
Deflazacort (0.9 mg/Kg – daily)*	Fewer mineralocorticoid effects; less weight gain	Cataracts; high-priced; unavailable in the Brazilian public health care system	2/year
Prednisone (0.75 mg/Kg – daily)**	Reasonable cost; available in the Brazilian public health care system	Higher risk of bone decalcification; more weight gain	2/year
Prednisone (5 mg/kg on Saturday and Sunday)	Low cost; available in the Brazilian public health care system	Higher risk of bone decalcification; more weight gain	2/year
Prednisolone (0.75 mg/Kg – daily)***	Low cost	Higher bone decalcification risk; more weight gain	2/year
Prednisolone (0.75 mg/Kg – 10 days on and 10 days off) ****	Low cost; fewer side effects	Higher risk of bone decalcification; more weight gain	3/year

Notes: Voting results: *45.5% neither agree nor disagree; 27.3% disagree; **27.3% agree; 27.3% neither agree nor disagree; ***27.3% agree; 27.3% neither agree nor disagree; and ****27.3% neither agree nor disagree; 45.5% disagree. Reproduced with the permission of *Arquivos de Neuro-Psiquiatria* . 3

In a population-based study, Kim et al. 22 have described weight gain, behavioral changes, and loss of ambulation as the main reasons for CS discontinuation. However, the side effects of CSs may be better addressed with a dose reduction of 25% to 33% followed by an early reassessment (1 month). If significant loss of function is observed, a new dose increment is indicated combined with shorter periods for clinical assessments (two- to three- month intervals). 2

At last, as future directions regarding the use of CSs for DMD patients, evidence from a posthoc analysis of the control group of the ACT DMD trial, 23 a meta-analysis of control groups from two different trials, 24 a real-world cohort, 25 and a clinical trial 26 suggest that deflazacort may result in better outcomes and less adverse events than prednisone/prednisolone. A double-blinded, randomized clinical trial 27 including 196 boys aged 4 to 7 years, designed to compare daily prednisone, daily deflazacort, and prednisone ten days on and ten days off, recently published its results. The daily regimens were more effective, regardless if on prednisone or deflazacort 27 (level of evidence: 2; class of recommendation: B)

Another interesting trend is related to synthetic CSs, such as vamorolone. 28 The open-label extension thirty-month study showed sustained slower progression in motor function tests, with fewer adverse events than CS. 29 Despite this promising scenario, a head-to-head clinical trial is important. Furthermore, the drug must be submitted for registration approval as well as made available in public health care to really become an option to CSs in Brazil.

## REHABILITATION

### Have new publications better defined inspiratory and expiratory muscle training?

The theoretical rationale for respiratory muscle training in patients with neuromuscular diseases is that it can improve muscle strength and endurance. Strengthening the inspiratory muscles, directly or indirectly, through the training of the expiratory muscles could, a priori, delay the need for ventilatory support, among other outcomes. However, the benefit of respiratory-muscle training in patients with DMD is still controversial. A recent meta-analysis 30 could not reach a conclusion about the effects of respiratory-muscle training.

### Respiratory assessments: When and how often Are They performed?

See systemic care.

### Motor assessments: When and how often Are They performed?

The assessments with motor scales should be performed every six months, and, with some exceptions, every four months (level of evidence: 5; class of recommendation: D; expert opinion: 50% strongly agree; 43.8% agree). 2 31

For the phase-1 motor assessment, developmental motor scales on motor milestone are the follow-up parameter (level of evidence: 5; class of recommendation: D). 2 Therefore, the Bayley Scales of Infant and Toddler Development – Bayley III assesses patients aged from 1 month to 3 years and a half in 5 domains (cognitive, linguistic, motor, socioemotional and adaptive behavior); it is suitable for research, but restricted to the clinical practice due to the extensive application time. 32 The Alberta Infant Motor Scale (AIMS) is usually part of the daily routine of physical therapists. It serves as a warning to evaluate these children and it is an exploratory, simple and easy tool for developmental assessment of children up to the age of 18 months or until they acquire independent gait. 2 In practice, due to the need to monitor the typical motor development of a child, the Brazilian Ministry of Health already provides essential attention booklets containing warnings regarding the motor function milestones at each age group and guidance on finding motor deficits. 33 Development monitoring warnings in the child's immunization record (vaccination booklet) can serve as a guide for families. 34 In phases 2 and 3, the timed tests, such as walking/running ten meters, rising from supine to standing, and climbing four steps, have an important predictive value to monitor the evolution and loss of gait. 35 36

In phases 4 and 5, in wheelchair-bound DMD patients, the function of the upper limbs is the essential motor assessment. The last Brazilian consensus ^4^ indicated the PUL scale, version 1.2. There is an update to version 2.0 with a conversion algorithm from version 1.2 to 2.0. 37 The Brooke scale rating system is easy and quick to apply, reflecting the functional level of DMD patients. 38 39 As for trunk control, there is a good correlation between the function of the upper limb and the Vignos Scale. 40 41

### What therapy is recommended in each stage of DMD?

Streching together with orthotics and alignment devides together help to delay ankle foot deformity. 31 Contracture prevention is an action combined with orthotics/alignment devices and stretching that, alone, does not benefit from preventing contracture (level of evidence: 2, class of recommendation: B). 42 Ankle–foot orthosis (AFO) should be used at night for ambulant DMD boys (phases 2 and 3). During the day, it is recommended to use insoles for pronated feet or supramalleolar (“small”) orthosis to align the medial and lateral malleolus while enabling movement of the tibiotarsal muscles for dorsiflexion (level of evidence: 5; class of recommendation: D; expert opinion). 31 When the patient becomes bound to a wheelchair, orthoses should be used during the day (level of evidence: 5; class of recommendation: D; expert opinion). 31 In the transition phase from ambulation to non-ambulation (phases 3 to 4), the use of a device that locks the knees in extension, such as the extensor pads or knee–ankle foot orthosis (KAFO), will be necessary, facilitating the therapeutic gait and delaying the gait loss (level of evidence: 5, class of recommendation: D; expert opinion). 31

Movements that involve eccentric contractions should be avoided as a prescribed form of exercise, like going downstairs, jumping on a trampoline, or going down ramps. (level of evidence: 5; class of recommendation: D; expert opinion). 43 44 45 A light to moderate isometric resistance exercise program in DMD boys with independent ambulation is safe, improving strength and function (level of evidence: 2; class of recommendation: B). 46 There is a need to perform mobility/flexibility activities for all joints and the trunk at all stages of the disease (level of evidence: 2; class of recommendation: B). 47

The present consensus does not recommend whole-body vibration (WBV), considering that there is no evidence of added benefit, 48 neither the PEDIASUIT/THERASUIT programs in DMD. Given the description of the technique and the care in prescribing exercises for DMD, long-term therapies that lead to fatigue should be avoided (level of evidence: 2; class of recommendation: D).

### What are the updates on muscle strengthening and exercises in DMD?

Further evidence is required to recommend strengthening exercises for DMD patients. The evidence available suggests that strengthening and aerobic exercises alone may have little or no effect on DMD (level of evidence: 5D, class of recommendation: D; 44 expert opinion:43.8% agree; 18.8% neither agree or disagree).

### Is there an indication for telemedicine and remote assessment in DMD?

Telerehabilitation is acceptable to DMD patients and caregivers. Instructions on activities can be delivered by means of video devices. The exercises are performed with the help of the caregiver (level of evidence: 2; class of recommendation: C; 49 expert opinion: 56.3% strongly agree; 25% agree).

### Are physical activities indicated, and Do They Offer benefits in DMD?

Participation in physical activities benefits people with disabilities and meets their social and psychological needs, aiming for a better quality of life. This is a recommendation of the present consensus (level of evidence: 3; class of recommendation: B; 50 expert opinion: 81.3% strongly agree; 12.5% agree).

### What Are the needs of an adult DMD patient?

The transition period should be sought for all young people: employment, accommodation, community life, financial independence, peer life, sexuality, leisure, daytime entertainment programs, art therapy, specialized nursing homes, and support services for the family (level of evidence: 3A; class of recommendation: A; 51 expert opinion: 75% strongly agree; 18.8% agree).

## SYSTEMIC CARE

The most recent advances on this topic described are as follows. 52 53 54 55 56 57

Regarding systemic care, recommended for all stages:

### General care (nutritional and immunizations)

- Monitor height and weight (level of evidence: 5D; class of recommendation: D); 2 4
- Monitor and follow the national recommendations for ferrous sulfate and vitamin D prophylaxis/treatment ( Table 3 ) (level of evidence: 5D; class of recommendation: D); 4 58
- Follow-up annually with blood measurements of calcium and 25-OH vitamin D, and blood measurements at baseline of calcium, 25-OH vitamin D, phosphorus, magnesium, phosphatase, and parathyroid hormone are important. 2 56 Supplement as needed ( Table 3 ) (level of evidence: 5D; class of recommendation: D);
- Special vaccines are recommended ( Table 3 ). 4 Vaccination against severe acute respiratory syndrome coronavirus 2 (Sars-COV-2) must follow the recommendations of the Ministry of Health (level of evidence: 5D; class of recommendation: D; expert opinion: 69.2% strongly agree; 30.8% agree).

**Table 3:** Updates on medications, supplements, and vaccines for Duchenne muscular dystrophy (DMD) subjects

Drugs and vaccines	
Vitamin D 58	Prophylaxis in boys with DMD (> 1 year): 1,000 IU/day; Treatment (deficiency if 25-OH-vitamin D levels < 12 ng/ml): 1-12 years: 3,000–6,000 IU/day (12 weeks); > 12 years: 6,000 IU/day (12 weeks). After treatment, keep regular use of prophylactic doses.
Calcium carbonate (1g = 400 mg of elemental calcium)	Supplement if there is vitamin D deficiency 40-80 mg/kg/day of elemental calcium, oral, 8/8h (4 weeks).
Ferrous sulphate 60	Oral iron (dose of 3 to 6 mg of elemental iron/kg/day), fractionated or in a single dose, for 6 months or until replacement of body stores confirmed by normalization of hemoglobin, MCV, HCM, serum iron, transferrin saturation, and serum ferritin.
Biphosphonates 55 56 57	Measure cystatin-C or, if unavailable, creatine clearance; Oral use is not recommended; Pamidronate: 6-9mg/kg/year IV every 3-4 months; Zoledronic acid: 0.1 mg/kg/year every 3 to 4 months.
Pneumococcal conjugate vaccine (Pn10 or Pn13)	2, 4, and 12 months.
Pneumococcal polysaccharide vaccine (Pn23)	One dose after the age of 2 years.
Influenza vaccine	Annual dose from 6 months of age.
Yellow fever vaccine	Contraindication if the subject is using prednisone 2 mg/kg/day or equivalent.
Angiotensin-converting enzyme inhibitors	Preferred use of enalapril: 0.1 to 0.5mg/kg/day in 1 to 2 doses; If not, an alternative is captopril: 6 years to adult age: 12.5 mg/dose, 2x/day; adults: 25 mg/dose, 2-3x/day.
Beta-blockers	Preferred use of carvedilol: 0.1 to 0.4 mg/kg/day (maximum of 1 mg/kg/day), 2x/day.
Other drugs used in cardiac management	Espironolactone: 1-3 mg/kg/day; Ivabradine: 2.5 mg 2x/day until 7.5 mg 2x/day; 52

Abbreviations: MCV, mean corpuscular volume HCM is wrong; MCH, Mean corpuscular Hemoglobine.

### Cardiological care

A cardiological evaluation is recommended since the first consultation, preferably with a cardiologist (expert opinion: 76.9% strongly agree; 15.4% agree). Annual assessment with electrocardiogram (ECG), and either cardiac magnetic resonance (MRI) or echo-strain or convencional echocardiogram are recommended (expert opinion: 46.2% strongly agree; 53.8% agree). It is recommended to start cardioprotection since the first visit if the patient is younger than 10 years of age. However, new studies 59 suggest that it could be beneficial even earlier or at any time if the tests are abnormal (expert opinion: 30.8% strongly agree; 46.2% agree). For this purpose, preferably use enalapril associated with spironolactone ( Table 3 ). If there are signs of heart failure, an aldosterone inhibitor associated with angiotensin-converting enzyme (ACE) inhibitors is recommended ( Table 3 ) (expert opinion: 30.8% strongly agree; 38.5% agree).

### Respiratory care

- Recommendation: caregivers and patients should be aware and prepared for possible respiratory complications. It is strongly recommended that patients and caregivers be familiar with lung-volume-recruitment maneuvers and manual cough assistance ( Appendix 1 ) (level of evidence: 2B; class of recommendation: B); 2 6 and
- Consider ventilatory support in asymptomatic patients but with forced vital capacity (FVC) < 50% of predicted, maximal inspiratory pressure < 60 cmH2O, polysomnography with non-invasive measurement of CO2 ≥ 50 mmHg for more than 2% of the total sleep time, the elevation of at least 10 mmHg between the waking CO2 value and the value obtained during sleep for more than 2% of the total sleep time, SpO2 < 88% for more than 2% of the total sleep time in the absence of identifiable respiratory events or for 5 consecutive minutes (criteria commonly used in services in which polysomnography is not available) 2 (level of evidence: 5; class of recommendation: D).

### Emotional support

It is recommended to offer emotional support and identify and manage cognitive, educational, and emotional issues 2 4 (level of evidence: 5; class of recommendation: D).

The recommendations on systemic care in DMD, specific to each stage, can be seen in Table 4 .

**Table 4:** Recommendations on systemic care in Duchenne muscular dystrophy (DMD), specific for each stage

	Stage 2	Stage 3	Stage 4	Stage 5
**General care**		**Outline a transition plan with the patient and family**		
		Attention to fluid intake and constipation		
		Consider swallowing assessment 61		
				Assess nutritional and swallowing status. Consider gastrostomy
**Bone health and orthopedic management**		Monitor scoliosis on every medical visit (physical inspection) and on regular imaging. Provide adaptation of the wheelchair. Eventually, in some patients, surgical spinal fusion may be needed 2 4 56 (level of evidence: 5D; class of recommendation: D)		
		To screen for osteopenia, thoracolumbar spine radiography (ask for Genant Score Calculation to be calculated by a radiologist) is recommended biannually when using corticosteroids and less frequently in those patients not receiving steroids or at any time in axial pain. If available, a dual-energy x-ray absorptiometry scan can help monitor the bone over time 4 56 (level of evidence: 5D, class of recommendation: D; expert opinion: 38.5% strongly agree; 46.2% agree)		
			If bisphosphonates use is required, measure cystatin-C before each infusion (risk of renal failure) (expert opinion: 38.5% strongly agree; 38.5% agree). The use of alendronate or oral bisphosphonates is not recommended ( Table 3 ) 2 56 (level of evidence: 5D; class of recommendation: D; expert opinion:.1% agree; 38.5% disagree)	
**Respiratory management**	Annual pulmonary function (spirometry, maximal respiratory pressures, and peak cough flow) (level of evidence 2B, class of recommendation B)			
	Implementation of proactive techniques for pulmonary recruitment				
	Consider type-1 polysomnography in patients with signs of sleep-disordered breathing, overweight, and those who fail to perform lung function tests well.				
		Assessment of the presence of dyspnea in different situations, dysphagia, and signs and symptoms of alveolar hypoventilation*		
			With loss of ambulation, perform a pulmonary functional assessment at least twice a year, with spirometry, maximal respiratory pressures and peak cough flow, oximetry, and assessment of CO2, or gasometry.	
			Annual polysomnography with CO2 measurement	
			Consider ventilatory support	
				Ventilation by tracheostomy has been an increasingly controversial topic and should be a decision shared with the patient and their family (level of evidence: 5D; class of recommendation: D)
**Cardiac management**		Annual Holter monitoring starting at age of 10 years in patients with normal electrocardiogram and starting at any age in the presence of a cardiac arrhythmia		
				Consider heart transplantation (patients with good lung function and mild peripheral muscle change)
				Consider implantable cardioverter defibrillators (ICDs) if a symptomatic and complex arrhythmia is present
**Mental health**	Attention to learning difficulties in school and cognitive and behavioral comorbidities			
				Special attention to mental health

Note: * Signs and symptoms of hypoventilation: dyspnea at daily activities; orthopnea; poor sleep quality with complaints such as insomnia, nightmares, frequent waking up; nocturnal or morning headache; fatigue or excessive daytime sleepiness; energy loss; reduced intellectual performance; depression; loss of appetite, nausea; autonomic dysfunction; and recurrent respiratory infections.

### Respiratory emergency management (level of evidence: 5; class of recommendation: D; expert opinion) 61

- It is recommended that patients have a pulse oximeter at home for the SpO2 assessment, especially in infectious respiratory exacerbations or unexplained ventilatory worsening;
- If SpO2 is < 95% in normal conditions (room air), in the presence of an infectious disorder, the patient and family should be instructed to increase cough-assistance maneuvers and, also, the frequency of lung-recruitment techniques. In some subjects, the use of non-invasive ventilation may also be necessary;
- If the condition persists or worsening signs are present, the doctor should be contacted;
- When refered to an emergency unit, patient should bring his own ventilatory equipment and manual or mechanical cough aid device (if they are in use); and
- It is essential to alert the care team that the isolated use of oxygen is not indicated.

Appendix 2 contains a suggestion of guidance for patients in emergencies. The patient and their family or caregivers should discuss these situations in advance.

### Guidelines for an indication for a surgical approach

- The use of inhaled anesthetic drugs or depolarizing neuromuscular blockers (succinylcholine) must be avoided. When exposed to these agents, DMD patients are at a potentially fatal risk of rhabdomyolysis and hyperkalemia;
- Evaluations by the cardiologist and pulmonologist are recommended before any surgical procedure. Anesthetists should be aware of the potential cardiologic and respiratory complications occurring intraoperatively and postoperatively;
- The proactive breathing techniques must be reviewed, and if the patient and family can perform them, even if there is still no indication of doing so regularly; and
- Non-invasive ventilation may also be necessary for the postoperative period. It should already be discussed with the patient and implemented before surgery, especially in subjects with an FVC < 50% of the predicted value, and it is mandatory for patients with an FVC < 30% than expected (level of evidence 2B; class of recommendation: B). 62

## NEW PERSPECTIVES

In our previous paper, Araujo et al. 3 listed the promising therapies in clinical trials by the time of that review. Targeted to the production of a more functional dystrophin, medications were being developed, to approach specific mutation types, the exon skipping agents and the readthrough premature stop codon drug. Patients with deletions amenable to the skipping of 51 could be potential candidates for either drisapersen or eteplirsen, and those with a nonsense mutation for ataluren.

More studies have been published on this topic, and we have new perspectives. Certain interventions have reached phase-3 clinical trials, have had positive results, and have been approved by regulatory agencies.

### Exon-skipping agents

Since our last review, the results on drisapersen of a phase-3 study 63 have been published: a subgroup achieved significant improvement on the 6 minute walking test, some adverse events were found, and the drug has been withdrawn from the market. Suvodirsen, a drug also addressed for amenable exon-51 skipping, was discontinued not due to safety reasons but to a non-significant increase in dystrophin levels. 64

Other exon-skipping agents have shown an increase in dystrophin levels in clinical studies with small samples. For exon-51 skipping with eteplirsen, extension studies, comparisons with natural history, and longer-term follow-up with pulmonary, cardiac, and motor functions show potential clinical benefits 65 66 67 68 (level of evidence: 2B). For patients with amenable exon-53 skipping, golodirsen has a good safety profile, 69 but clinical improvement should be further addressed. The same goes for viltolarsen, also an exon-53 skipping agent, with favorable safety in a phase-2 study, 70 and for the Japanese morpholino antisense-nucleotide. 71 Finally, for amenable exon-45 skipping, casimersen in a phase-1/2 study 72 has been proved to increase dystrophin and have good safety.

The US Food and Drug Administration (FDA) approved eteplirsen/exondys in 2016, 73 godolirsen/viltolarsen in 2019, 74 casimersen/amondys in 2021, 75 and vitolarsen/viltepso in 2020, 76 based on the surrogate endpoint: the increase of dystrophin. Confirmatory clinical data is currently being obtained (see Appendix 3 for a list of phase-3 clinical trials). The European Medicines Agency (EMA) refused the marketing authorization for eteplirsen/exondys in 2018 by considering the small sample presented and asking for more clinical outcome results in clinical trials. 77

### Nonsense readthrough therapy

Phase-3 results on ataluren, an oral drug designed to enable full-length dystrophin protein production, have been published. The multicentric, randomized, double-blinded, placebo-controlled trial included 228 DMD ambulatory boys aged 7 to 16 years. Change in disease progression was more evident in the prespecified subgroup of patients with a baseline 6MWD of 300 m to 400 m. Ataluren was generally well tolerated 78 (level of evidence: 1B).

A further meta-analysis of phase-2 and -3 clinical trials supports the previous evidence. 79 Finally, additional postmarketing on a registry of 200 DMD patients, a real world evidence, was compared to the data from a natural history study. Through the propensity score methodology, samples were matched, and ataluren benefit was confirmed. Subsequent loss of ambulation with statistical significance in those treated with ataluren, and a trend toward subsequent loss of pulmonary and cardiac function in a broader age range than those in the clinical trials corroborate previous evidence.

The EMA decided that ataluren/translarna's benefits were more significant than its risks, and the drug received conditional marketing authorization in 2014. In 2021, a renewal of the authorization was granted. 81

In Brazil, ANVISA has approved ataluren/translarna in 2019 and recently extended the age range for its use from 2 years on. 82

### Gene therapy

Gene therapy uses viral vectors, to deliver to the cells new gene material. Because the DMD gene is very large, a smaller version of this gene has to be used. Trials are ongoing for phase-3 gene transfer, using different vectors and variable smaller DMD gene versions (see Appendix 3 for the list of clinical trials).

### Others

Other targets that do not involve dystrophin production or the different aforementioned steroid therapies are also being explored: TAS-205, a drug that blocks hematopoietic prostaglandin D synthase, could limit inflammation in DMD and subjects are currently being recruited for a phase-3 study. Myostatin inhibition or blockade, aiming to improve muscle bulk and function, has been explored but discontinued, either because of adverse events or for not showing as effects as good as planned. 83

The working group recommendation on new perspectives is that, in order for a drug to be widely used in Brazil, it must not only have its registration approved by ANVISA but also be available for public health care after inclusion by the Brazilian National Committee for the Inclusion of Technology in the Unified Health System (Comissão Nacional de Incorporação de Tecnologias no Sistema Único de Saúde, CONITEC, in Portuguese) and or listed by The National Supplementary Health Agency (Agência Nacional de Saúde Suplementar, ANS, in Portuguese) to be reimbursed by health insurance companies (expert opinion: 45.5% strongly agree; 36.4% agree).

## Figures and Tables

**Table:** 

Test name	Equipment	Target measure
PEMax	Manometry	Expiratory muscle strength
PIMax	Manometry	Inspiratory muscle strength
Peak flow	Peak flow	Assessment of the mobilization efficacy of cough and bronchial secretion
SNIP	Manometry	Inspiratory muscle strength
VFC	Spirometry	Pulmonary capacity assessment
PEF	Spirometry or peak flow	Indirect pulmonary capacity assessment through forced expiratory flow

## Notes

Appendix 1: Main respiratory function measures Abbreviations: PEF, peak expiratory flow; PEMax, maximum expiratory pressure; PIMax, maximum inspiratory pressure; SNIP, inspiratory nasal sniff pressure; VFC, vital forced capacity. Adapted from: Boentert M, Prigent H, Várdi K, Jones HN, Mellies U, Simmonds AK, Wenninger S, Cortés EB, Confalonieri M. Practical Recommendations for Diagnosis and Management of Respiratory Muscle Weakness in Late-Onset Pompe Disease Int. J. Mol. Sci. 2016, 17, 1735. Techniques for DMD patients Pulmonary recruitment maneuver To reach maximum pulmonary insufflation, active or passive using:

A. Manual resuscitator;
B. Glossopharyngeal respiration; and
C. Volume ventilator.

The patient must retain the volume of air until maximum insufflation is achieved, and then exhale or cough. If collaboration is not possible, passive insufflation is conducted. Coughing aid Cough can be aided with manual thoracic compression ideally combined with abdominal compression. Mechanical cough can be attempted with the appropriate equipment, if the peak flow is < 160 L/min or if thoracic compression is contraindicated.

## Notes

Appendix 2
**EMERGENCY ALLERT**
I have Duchenne muscular dystrophy, a neuromuscular disease that has consequences on pulmonary ventilation because of muscle weakness. This turns cough and secretion clearance inefficient. In an acute clinical condition, as respiratory infection, I become more vulnerable. What should be done when I find myself in such a situation, with or without oxygen desaturation:

A. Oxygen should not be administered in isolation (risk of CO2 retention and decrease in level of consciousness);
B. No-invasive bilevel ventilatory support might be needed. If hypoxic oxigen can be offered once ventilation is installed; and
C. Contact assistant health care provider for more information.

## Notes

Appendix 3 Phase-3 exon-skipping trials for Duchenne Muscular Dystrophy: A study to compare safety and efficacy of a high dose of eteplirsen in participants with Duchenne Muscular Dystrophy.
https://clinicaltrials.gov/ct2/show/NCT03992430?term=eteplirsen&cond=DMD&draw=2&rank=7
Study of SRP-4045 (Casimersen) and SRP-4053 (Golodirsen) in Participants With Duchenne Muscular Dystrophy.
https://clinicaltrials.gov/ct2/show/NCT02500381?term=golodirsen&cond=DMD&phase=2&draw=2&rank=2
Study to Assess the Efficacy and Safety of Viltolarsen in Ambulant Boys With DMD.
https://clinicaltrials.gov/ct2/show/NCT04060199?term=viltolarsen&cond=DMD&draw=2&rank=3
